# Cognitive Deterioration and Associated Pathology Induced by Chronic Low-Level Aluminum Ingestion in a Translational Rat Model Provides an Explanation of Alzheimer's Disease, Tests for Susceptibility and Avenues for Treatment

**DOI:** 10.1155/2012/914947

**Published:** 2012-07-30

**Authors:** J. R. Walton

**Affiliations:** ^1^Faculty of Medicine, University of New South Wales, Sydney, NSW 2052, Australia; ^2^Clinical Outcomes Research, St George Hospital, Kogarah, NSW 2217, Australia

## Abstract

A translational aging rat model for chronic aluminum (Al) neurotoxicity mimics human Al exposure by ingesting Al, throughout middle age and old age, in equivalent amounts to those ingested by Americans from their food, water, and Al additives. Most rats that consumed Al in an amount equivalent to the high end of the human total dietary Al range developed severe cognitive deterioration in old age. High-stage Al accumulation occurred in the entorhinal cortical cells of origin for the perforant pathway and hippocampal CA1 cells, resulting in microtubule depletion and dendritic dieback. Analogous pathological change in humans leads to destruction of the perforant pathway and Alzheimer's disease dementia. The hippocampus is thereby isolated from neocortical input and output normally mediated by the entorhinal cortex. Additional evidence is presented that Al is involved in the formation of neurofibrillary tangles, amyloid plaques, granulovacuolar degeneration, and other pathological changes of Alzheimer's disease (AD). The shared characteristics indicate that AD is a human form of chronic Al neurotoxicity. This translational animal model provides fresh strategies for the prevention, diagnosis, and treatment of AD.

## 1. Introduction

More than 36 million people are currently living with dementia worldwide [1] and about 75% of this population, that is, 27 million people, are estimated to be affected by Alzheimer's disease (AD) [2]. A comparison of the health of older persons, described in the monograph *Old Age* published in Cambridge in 1889 [3] and papers published in the *British Medical Journal* between 1886 and 1889, with the health status of older persons today, raises the possibility that AD is a modern disease that has developed from altered living conditions associated with the industrialization of society [4].

The monograph examines the results of a health survey carried out by the British Medical Council where British general practitioners systematically assessed the health of their oldest patients during the mid-1880s. The study group consisted of almost 900 subjects, aged 80 years and older, including 74 centenarians. The monograph author states “[Dementia, the] saddest state of all, was witnessed only in two of our centenarians. …Indeed, the brain in many held out as well or better than other organs” [3]. By way of contrast, a study conducted in 2000 found that 88% or 15 of all the 17 centenarians living in three Dutch towns, with populations of 250,000 or more, had dementia. The other two centenarians could not be examined [5].

Upon describing the first known case with this disease, Alzheimer himself wrote, “The case presented even in the clinic such a different picture, that it could not be categorised under known disease headings, and alsoanatomically it provided a result which departed from all previouslyknown disease pathology” [6].

We developed an aging rat model for chronic aluminum (Al) neurotoxicity [7, 8] to learn what, if anything, would happen if rats were fed human-relevant Al levels over a prolonged time. We discovered that the model replicates early stages of AD and more severe cognitive deterioration, thus helping to explain AD origin and progression.

## 2. An Al-Inducible Animal Model for Chronic Aluminum Neurotoxicity/AD

Outbred wild-type male Wistar rats were used for the model, which requires survival well into old age since chronic Al neurotoxicity and AD have a long prodromal period before becoming overt in older individuals. We chose males over females to improve uniformity of results in case any biochemical measurements were needed that could vary with the estrus cycle. The animals were allowed to live for their entire life span, undergoing normal brain aging to provide an improved model for studying neurodegenerative processes in the aged human brain. Old neurons are structurally and functionally different from young neurons, particularly with respect to sprouting and reinnervation capacities (e.g., [9, 10]).

The rats were trained at a young age to perform a rewarded continuous alternation T-maze task commonly used to assess memory function [11, 12]. They were given ten chances to choose alternate arms of the T-maze to achieve a maximum score of 100%. All trained rats were tested each week throughout their middle age and old age [7, 8]. Maze testing continued until the rats began to show marked evidence of physical decline.

From 6 months of age, the rats were fed 20 g per day of a low protein/low fat maintenance feed, containing 9 ppm Al, to standardize the amount of Al they received from their feed. This amount of feed was sufficient to maintain their body weights (bw) at a healthy level of 500 g ± 50 g. The feedings were provided twice weekly to motivate the rats for their weekly exercise in the T-maze and to ensure they drank some water between feedings. They were routinely given ultrapure HPLC-grade water without detectable Al [7, 8].

Exposure to additional Al was delayed until the rats entered middle age to give their brains ample time for normal development. Rats in the pilot study [7] were randomly assigned at 16 months to two groups; one that consumed only the Al contained in their feed and the other a high dose as described for the main study. Rats in the main study [8] were randomly assigned at age 12 months to three groups that consumed low, intermediate, and high Al levels in amounts equivalent to total dietary Al levels consumed by Americans from their food, water, and Al additives [13]. The additional Al was added to the drinking water of the intermediate and high dose groups to increase the Al concentrations of their water to 2 mg/L and 20 mg/L. The rats' average water consumption was 30 mL per day.

The low Al group had a total daily dietary Al intake of 0.4 mg/kg bw derived entirely from their feed. The intermediate group had a total Al intake of 0.5 mg/kg bw/day and the high group consumed 1.7 mg Al/kg bw/day. The only treatment difference was the amount of total dietary Al the three rat groups routinely consumed from their feed and water throughout middle age and old age [8].

Rats in the three treatment groups achieved similar mean performance scores of 70–100% for choice accuracy on the T-maze task during middle age (12 months up to 24 months). Rats in the low, intermediate, and high Al groups lived for 31.1, 32.4, and 30.5 months, respectively (*P* < 0.44). The average lifespan for all rats in this study was 31.3 months [8]. This age is approximately equivalent to 91 human years, given that Wistar rats age about 35 times faster than humans [14]. Routine Al ingestion at these levels showed no significant effects on the animals' kidney and liver functions [8].

## 3. Chronic Al Exposure in Humans

**Figure 1:**
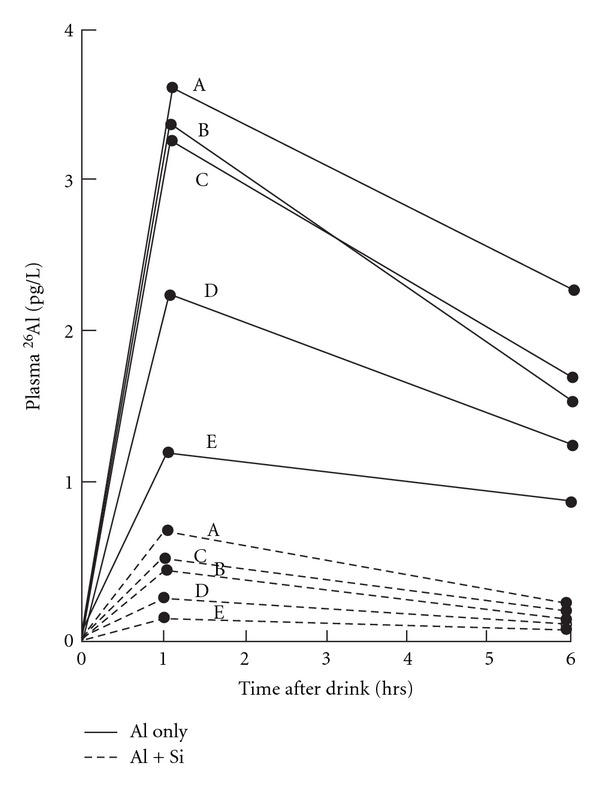
Some humans absorb more Al than others. Plasma ^26^Al levels in humans who drank ^26^Al with orange juice, and ^26^Al with orange juice plus silica. Silica lowers the amount of Al absorbed. The different individuals exhibit a spread of plasma Al values from (A) to (E), the highest being approximately three-fold times greater than the lowest. Note that ranking of subjects' plasma values is almost in the same order on both occasions. Reproduced from [19] with permission from Elsevier.

Al is a neurotoxicant without any useful biological function that more readily enters the brain than is able to leave. Hence, Al increases in the brain as the age rises [15–18]. Some human populations are more at risk than others for chronic Al neurotoxicity because humans, like rats, show wide variability in the amounts of Al they absorb from a standardized Al dose (e.g., Figure 1) [19].

A major route of Al exposure for humans is from Al salts in the form of dietary additives according to the World Health Organization [20]. Al salts are added to commercially prepared foods for a variety of reasons: for food coloring (Al serves as a mordant, binding food dyes to foods), as anti-caking agents in powdered foods, cheese-melting agents, a rising agent in cakes and other baked goods, pH adjusting agents, thickening agents, pickling agents, carriers, meat binders, emulsifiers, stabilizing agents, buffers, dough strengtheners, sweeteners, texturizers, gravy/sauce thickeners, curing agents, and hardening agents (e.g., for candied fruits). Al is added to urban water supplies and some bottled waters as a clarifying agent to give the water a crystal-clear appearance (reviewed in [13, 21]). Experiments with the ^26^Al isotope and accelerator mass spectrometry have shown that measurable amounts of Al can enter rat brains after they swallow a minute amount of Al equivalent to that contained in a single glass of alum-treated drinking water [22–24].

In 2007, the Joint Food and Agriculture Organization (of the United Nations)/World Health Organization (FAO/WHO) Expert Committee on Food Additives reduced the provisional tolerable weekly intake (PTWI) recommended for humans from 7 mg Al/kg bw to 1 mg Al/kg bw [25]. Thus, the current PTWI equates to a weekly intake of 70 mg Al for an average human of 70 kg or 10 mg Al/day. Most humans routinely exceed this PTWI level for Al. One half of the American population is estimated to consume up to 25 mg Al/day, 45% between 25 and 95 mg Al/day, and 5% take in more than 95 gm Al/day in the form of Al additives in addition to 1–10 mg Al/day contained in fresh foods [13].

Nondietary sources of Al exposure include the following.

1. Vaccines where Al serves as the adjuvant. Injected Al bypasses the protective mucosal barrier of the gastrointestinal tract. Experimental research has shown that Al adjuvants have the potential to induce significant immunological disorders in humans [26]. Simulated vaccination in young mice produces an Al peak in their brain after 2-3 days [27].
2. Some topical applications, such as sunscreens and deodorants, contain Al. One publication found transdermal uptake of Al chloride on shaved mouse skin was greater in the hippocampus than oral Al uptake [28], thus contributing to the brain's Al burden.
3. Pharmaceuticals and medical applications. Considerable amounts of Al are contained in Al antacids, buffered aspirins, and some other pharmaceuticals [29]. Some medical treatments also utilize Al (e.g., alum bladder irrigations).

**Figure 2:**
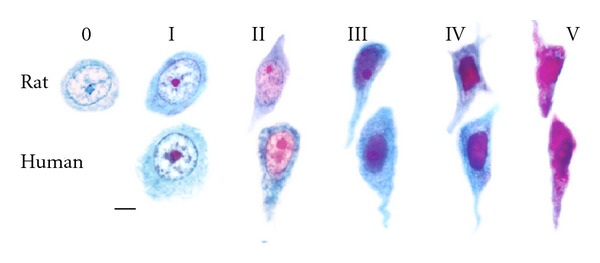
Staging of aged rat (upper row) and aged human (lower row) hippocampal CA1 neurons stained for Al [30, 31] show progressive Al accumulation accompanied by cytopathological change. Stage 0: the entire cell appears Al-negative and has normal morphology; this stage is not observed in the aged human specimens. Stage I: magenta nucleolus, no other staining for aluminum. Stage II: magenta nucleolus in pink nucleoplasm with visible chromatin; the cytoplasm is blue. Stage III: magenta nucleolus in an elongated or irregularly shaped purple nucleus. The cytoplasm is blue. Many apical dendrites from this stage onwards have a serpentine appearance. Stage IV: the magenta staining appears in the elongated nucleus which now shows less structural detail; the shrunken cytoplasm is still blue. Stage V: purple to magenta staining appears throughout the nucleus and cytoplasm. Cell shape is distorted and the axon and dendrites are disrupted. Magnification bar (MB) = 15 *μ*m, Reproduced from [31].

Human groups currently believed to be at most risk for chronic Al neurotoxicity are (1) older humans whose neurons have had sufficient time to accumulate Al to stages IV and V (Figure 2); (2) humans that regularly consume a diet of commercially prepared foods rather than fresh foods; (3) those with AD or; (4) Down's syndrome, who absorb Al considerably more efficiently; (5) those with mildly impaired kidney function who lack efficient Al removal; (6) those in families that have a tendency to develop AD, possibly because they have a genetic constitution that predisposes them to efficient Al absorption. Human groups at risk for chronic Al neurotoxicity are very similar to those with increased risk for AD.

To date, Al/iron chelation by desferrioxamine is the only treatment for AD that has been shown to slow the rate of deterioration in activities of daily life [32, 33].

## 4. Chronic Aluminum Neurotoxicity Results in Cognitive Deterioration in Aged Susceptible Individuals

Statistical analysis of the rats' test scores revealed that the rats of the main longitudinal study developed cognitive impairment in an Al dose-dependent manner [8]. None of the rats that consumed Al at the low end of the human dietary Al range obtained significantly lower performance scores in old age than in middle age. In fact, scores of the oldest rat, who survived to age 38 months, showed near-significant improvement in old age (*P* > 0.057). However, 20% (2/10) of the intermediate Al rats and 70% (7/10) of the rats that consumed Al at the high end of the human Al range had significantly lower scores in old age than in middle age [8]. These data validated observations made in the pilot study [7] that showed essentially the same results in a smaller number of animals. Middle-age and old-age performances of one rat that developed cognitive deterioration are shown in the accompanying QuickTime video clips of the Supplementary Material available online at doi:10.1155/2012/914947. 

The nine rats with statistically significant declines in their mean T-maze performance scores between middle age and old age also showed evidence of abnormal behaviors and signs, such as incontinence while in the T-maze, perseverative behaviors, head nodding, and seizures. Rats with low performance scores were apparently unable to recognize the spouts on their more upright water bottles during an overnight stay in metabolism cages. In contrast, the water levels decreased in bottles of metabolism cages containing rats that scored normally during the same time period [7]. The rats with abnormal behaviors and significantly lower mean T-maze performance scores in old age than middle age are referred to here as “cognitively deteriorated.”

Upon reviewing the performance scores at the end of the study we observed that most rats that developed cognitive deterioration made more mistakes than usual in their T-maze performances from age 27 months and onwards. By the time they were 28 months old, several had already attained mean scores in their old age that were significantly lower than in middle age. Eventually, rats with cognitive deterioration stopped performing altogether. One rat in the high Al group showed early performance impairment from age 20 months [8]. We surmised that this rat with early onset cognitive deterioration and the two rats that developed cognitive deterioration after consuming the intermediate Al dose level had some type of physiological difference in their ability to absorb Al that rendered them particularly susceptible to chronic Al neurotoxicity. All three rats exhibited unusually high plasma Al levels for their treatment groups.

## 5. Chronic Aluminum Neurotoxicity in the Aged Rats Parallels Specific Aspects of AD Neuropathology in Old Age

The rats were euthanized by Nembutal overdose when they became moribund with advanced age. Autopsies were performed and neuropathological assessments were carried out on their brains. Al accumulation in both rat and human neurons can be staged according to the criteria described in Figure 2. Most aged rat pyramidal neurons, like their aged human counterparts, at least stain for Al in the nucleolus while otherwise appearing normal (stage I) [30]. Many pyramidal neurons in the rats with cognitive deterioration stained for Al accumulation throughout their nucleus (stage IV). 

Several rat brains were completely serially sectioned, with every tenth section stained for Al [34], and surveyed to determine which brain regions were most prone to stage IV Al accumulation [8]. This examination disclosed that the main Al-affected brain regions in animals with chronic Al neurotoxicity include the entorhinal cortex, hippocampus, subiculum, septum, olfactory lobe, piriform cortex, temporal cortex, parietal cortex, frontal cortex, cingulate cortex, amygdala, substantia nigra, basal nucleus of Meynert, dorsal raphe nucleus, and locus coeruleus [8]. These are essentially the same brain regions affected by neurofibrillary tangles (NFTs) in Al-injected rabbit brains [35]. Interestingly, these are also the same brain regions particularly vulnerable to NFT formation and deterioration in AD [36, 37].

Computer-assisted cell counts on blinded rat brain sections enabled estimates to be made of the proportions of cells with high-stage (IV and V) Al accumulation in total pyramidal cell populations [31]. This type of analysis was made in association area 3 of the temporal cortex and in the entorhinal cortex of cognitively deteriorated rats and low Al (cognitively intact) controls. The entorhinal cortex was found to be the brain region most affected by chronic Al exposure in these animals. This is also the brain region most affected by NFTs in AD [38]. 

**Figure 3:**
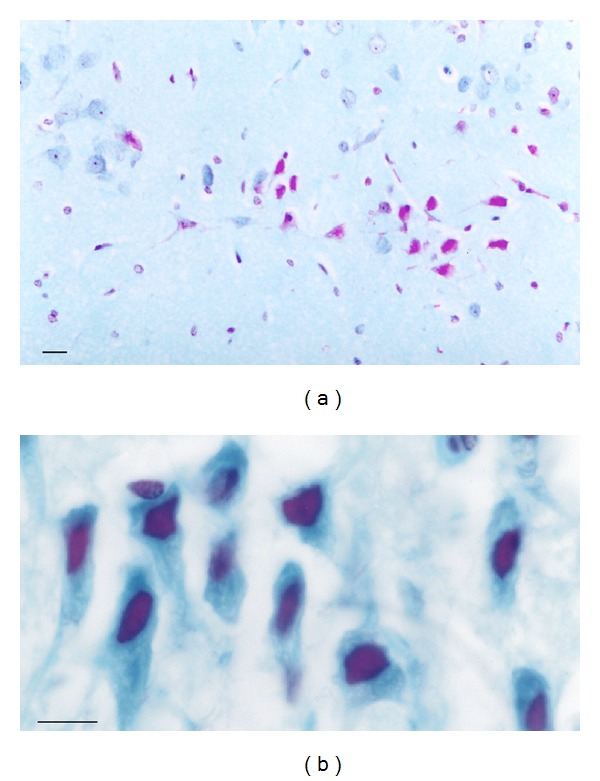
Entorhinal cortical cells with stage IV Al accumulation from rats with cognitive deterioration. (a) Low-magnification view of an island containing some cells with high-stage Al staining and others with normal morphology. (b) Island of stellate entorhinal cortical cells at high optical magnification. MB = 50 *μ*m.

Approximately 40% ± 7% of the pyramidal cells counted in association area 3 of the temporal cortex displayed stage IV Al accumulation in the cognitively deteriorated rats compared to 13% ± 3% in the low Al controls (*P* < 0.01). Approximately 60% ± 7% of the entorhinal cortex stellate and pyramidal cells counted in layer II and pyramidal cells in the superficial part of layer III from rats with cognitive deterioration had stage VI Al accumulation compared to 23% ± 7% in the low Al controls (*P* < 0.001) [31]. Examples of entorhinal cortical cells with stage IV Al accumulation are shown in Figure 3. The percentage of entorhinal cortical cells with stage IV Al accumulation correlated with the extent of change in the animals' T-maze performance scores between middle age and old age (*r* = 0.76; *P* < 0.0005) [31]. 

We carried out immunocytochemical studies to further probe how brain tissue of the rats with cognitive deterioration differed from the others. Immunostained hippocampal pyramidal cells of the rats with cognitive deterioration exhibited immunoreactivity for hyperphosphorylated tau, oxidative damage indicated by 4-hydroxynonenal adducts [39], elevated levels of amyloid precursor protein [40], and reduced levels of choline acetyltransferase in the basal nucleus of Meynert and striatum [Walton, unpublished observations].

The most striking finding with immunostains concerned microtubules. Microtubules normally provide structure to the cell body of the neuron, its axon and dendrites, and infrastructure for transport of nutrients, neurotransmitters, and organelles between the cell body and its most distant terminals [41]. Hippocampal sections from the low Al rat controls, immunostained with the anti-*α*-acetylated tubulin antibody, presented as continuous strips of hippocampal CA1, CA2, and CA3 pyramidal cells containing microtubules of uniform caliber and density. Microtubules were clearly visible in the soma and apical dendrites of pyramidal cells throughout the hippocampal formation, equivalent to those with stage I Al accumulation [31].

Hippocampal sections from the rats with cognitive deterioration contained some groups of immunostained hippocampal and cortical pyramidal cells that exhibited microtubules with irregular caliber and density in dendrites that appeared distorted and shriveled. Location of equivalent cells in these groups, on adjacent slides stained for Al, revealed the equivalent cells were at stage III Al accumulation [31].

**Figure 4:**
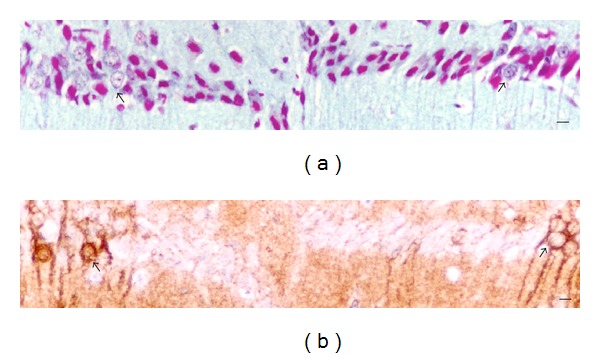
Al accumulation in a lesion of hippocampal CA1 cells in a rat with cognitive deterioration correlates with microtubule depletion. (a) The stage IV pyramidal cells in the center stain magenta for nuclear Al. Pyramidal cells with a normal appearance (arrows) are present along the margins of the lesion. (b) An adjacent section, immunostained for acetylated tubulin, demonstrates that cells within the lesion, corresponding to those with stage IV Al staining, are microtubule depleted. Other pyramidal cells at the margins of the lesion have a more normal appearance and clearly immunostain for microtubules (arrows). MB = 50 *μ*M. Republished from [31].

Other groups of pyramidal cells in the hippocampus and entorhinal cortex of rats with cognitive deterioration were equivalent to pyramidal cells with stage IV Al accumulation. The stage IV pyramidal cells appeared shrunken with small dense nuclei. Larger groups of these cells appeared in the form of a lesion amongst pyramidal cells with a more normal appearance (Figure 4). Adjacent sections, immunostained for acetylated-*α*-tubulin, demonstrated that the cells of the lesions lacked evidence of microtubules in their cell body [31]. Many also failed to immunostain for tubulin in their apical dendrite. One rat brain had a lesion of Al-rich microtubule-depleted cells in the subiculum instead of the hippocampal CA1 field.

Immunostaining was also carried out with an antibody immunostain for nonacetylated *β*-tubulin but this gave essentially the same result as the antibody immunostain for acetylated *α*-tubulin [31]. Again, cells adjacent to the lesion at lower stages of Al accumulation clearly demonstrated microtubules.

We also examined consecutive sections of AD brains, either stained for Al or immunostained for microtubules, and observed microtubule depletion in hippocampal cells with NFTs and in others with high-stage Al staining that lacked NFTs [31]. It was previously reported that AD cells with NFTs lack microtubules [42, 43] but the reason for this was unclear. Others had observed that AD pyramidal cells with early stage NFTs have low mRNA levels for cytochrome oxidase, a marker of mitochondrial energy metabolism and neuronal activity [44]. The structural and functional deficits of Al-rich cells without microtubules indicate they are unable to perform specialized neural functions.

**Figure 5:**
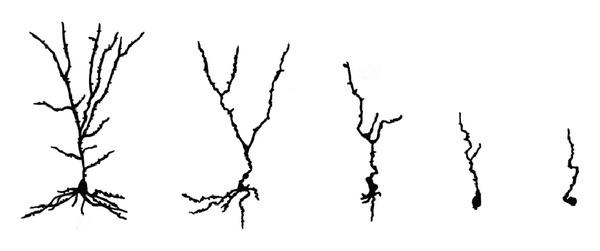
Camera lucida drawings of Golgi-stained AD hippocampal pyramidal cells that illustrate the process of dendritic dieback (left to right). The deteriorated cell at far right resembles some of the Al-rich microtubule-depleted cells in the hippocampal lesion of Figure 4(b). Redrawn from [45] with permission from Elsevier.

Cells without microtubules are also susceptible to dendritic dieback, culminating in loss of synapse density. This involves loss of dendritic spines, abnormal spindle-shaped swellings in dendrites, and progressive withering of the dendritic tree (Figure 5) [45]. Dendritic dieback has been shown to affect neocortical, as well as entorhinal cortical, hippocampal, and dentate granule cells of AD brain tissue [45] and hippocampal, subicular, and cortical cells of brains from Al-exposed laboratory animals [46, 47]. The cell's dendritic tree accounts for about 95% of its receptive surface area, [48] so massive dendritic loss may account for the cortical atrophy characteristic of severely affected AD brains.

**Figure 6:**
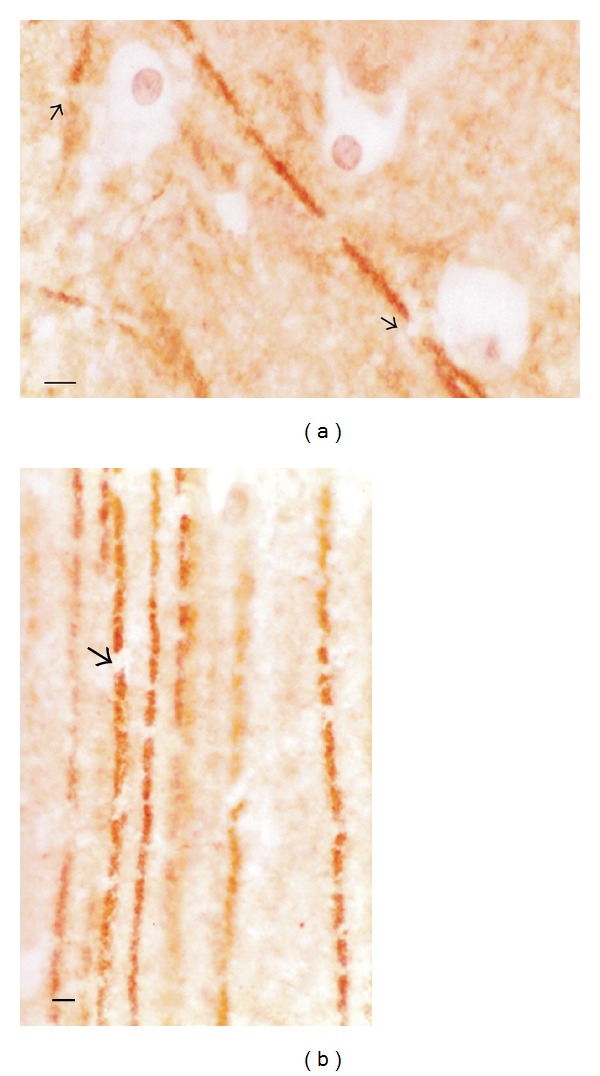
Dendritic dieback in AD brain revealed by immunostaining for microtubules. (a) Glial cells in the stratum radiatum appear to clip dendrites into segments. Portions of glial cell cytoplasm insert between dendritic segments (arrows). (b) Segmented dendrites of the stratum radiatum from an AD case. The arrow points to segmentation that interrupts dendritic microtubules. MB = 2.5 *μ*m. Republished from [31].

Glial cells in the stratum radiatum of the CA1/subicular zone of brains from rats with cognitive deterioration appeared to be clipping the damaged dendrites into nonfunctional segments (Figure 6) [31]. The dieback process is thought to occur slowly and have long-lasting effects [47]. The extensive pruning process of abnormal dendrites and their spines leads to reduction in hippocampal synapse density in rats with chronic Al neurotoxicity [31] as in humans with AD [49]. Thus, devastating consequences for neural function flow from Al-induced microtubule depletion in rat and human cells with stages IV or V Al accumulation [31]. Al concentrations around 100–250 *μ*M are typically found in aged brain cells affected with AD [50–54].

Brains of wild-type rats lack fully formed plaques and tangles for species-specific reasons, even in those with chronic Al exposure. Al involvement in plaque and tangle formation have been shown in transgenic mice and humans. One advantage of the present model is that it allows the study of cognitive deterioration without the complications of overlying plaque and tangle neuropathology. Secondly, the model demonstrates that cognitive deterioration in the model develops by a mechanism independent of plaques and tangles. The model also helps to explain why aged human cells in NFT-prone brain regions have a tendency to die. It has been observed, for example, that human cells that form NFTs can become enucleated by NFT overgrowth [30]. Aged pyramidal cells in the rat entorhinal cortex display high-stage Al accumulation and are clearly dysfunctional even though they lack both NFTs and clear evidence of large-scale cell death.

Entorhinal cortical cells of layers II and III are the cells of origin for the perforant pathway that conveys neocortical input to the hippocampus critical for memory processing [55]. Results from this translational animal model indicate that high Al accumulation in a large number of entorhinal cortical cells leads to cognitive deterioration in these rats, paralleling the cognitive deterioration that occurs in AD brains. At this point, it would be useful to briefly review the comparative anatomy of the entorhinal cortex and hippocampal formation in rats and humans.

## 6. Comparative Aspects of the Entorhinal Cortex and Hippocampal Formation in Normal Rats and Humans

The entorhinal cortex of the rat brain corresponds to the entorhinal cortex in humans, Brodmann's area 28. In humans, this brain region occupies the anterior portion of the parahippocampal gyrus [55]. The entorhinal cortex serves as a pivotal two-way station with the dual roles of funneling neocortical input into the hippocampal formation as well as funneling output from the hippocampal formation back to the neocortex [56]. A large number of neocortical regions project to superficial layers of the entorhinal cortex, including the olfactory, auditory, visual, and somatosensory cortices as well as multimodal areas and the amygdala. Projections from the olfactory lobe are particularly prominent in rat brain [57]. Layer IV of the human entorhinal cortex receives a heavy output from the hippocampal formation and reciprocates the perforant pathway by projecting widely back to the neocortex [56]. The entorhinal cortex in humans functions in basically the same ways as in rats although in the human brain the entorhinal cortex contains many more cells than in rat brain and is much more developed. Subareas of the entorhinal cortex and hippocampus differ in their proportions in human and rat brains [58]. 

The entorhinal cortex has 6 layers in rats and humans [59]. Layer I, the acellular molecular layer of the entorhinal cortex, is continuous with adjoining molecular layers of the neocortex and parasubiculum. Layer I contains terminal axonal branches from numerous neocortical inputs together with dendrites from the superficial neuronal layers of the entorhinal cortex. Layer II of the human and rat entorhinal cortex contains large stellate cells (70–80%), large multipolar neurons (3%), and modified pyramidal neurons (17–27%). The stellate neurons are located in cell clusters or “islands” separated by a dense reticular network [59–61]; they persistently generate rhythmic oscillations [62]. The superficial part of layer III contains pyramidal neurons.

**Figure 7:**
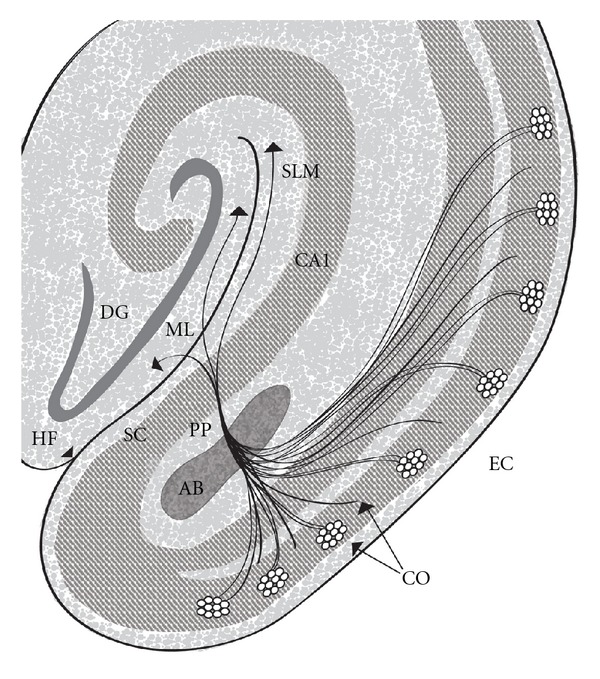
Schematic representation of the perforant pathway. The perforant pathway is similar for humans and rats apart from minor variations. (1) The cells of origin (CO) for the perforant pathway (PP) reside in layer II (shown as cell islands) and in the superficial part of layer III of the entorhinal cortex (EC). The cells of origin receive information from many cortical regions. (2) Axons of the cells of origin converge in the angular bundle (AB) from which the perforant pathway emerges. (3) Upon leaving the angular bundle the axons (4) diverge into fascicles known as the perforant pathway (PP) because they perforate the gray matter of the subicular cortex (SC) on their way to the hippocampal formation. (5) A contingent of fascicles enters the stratum lacunosum moleculare (SLM) of the CA1/subicular zone (CA1) and terminates on pyramidal cell dendrites. (6) More fascicles cross the hippocampal fissure (HF) (7) to enter the molecular layer (ML) of the dentate gyrus (DG) and terminate on distal dendrites of granule cells in the outer two-thirds of this molecular layer. Based on information contained in [63] with permission from John Wiley and Sons.

The cells of origin for the perforant pathway are stellate and pyramidal neurons of layers II and III. These cells give rise to axons that collect in an angular bundle and then project massively to the hippocampal formation in the form of distinct fascicles (Figure 7). These cells are more sparse in rats than in humans but are functionally similar [58]. Their axons perforate or pass through gray matter of the subiculum on the way to their terminal sites [56]. Some fascicles remain in the stratum lacunosum moleculare of the hippocampal formation where they terminate on distal apical dendritic branches of hippocampal CA1 and subicular pyramidal cells [56]. The largest contingent of fascicles crosses the hippocampal fissure to terminate in the outer two-thirds of the dentate gyrus molecular layer in a rigidly stratified manner on distal dendritic branches of dentate gyrus granule cells.

**Figure 8:**
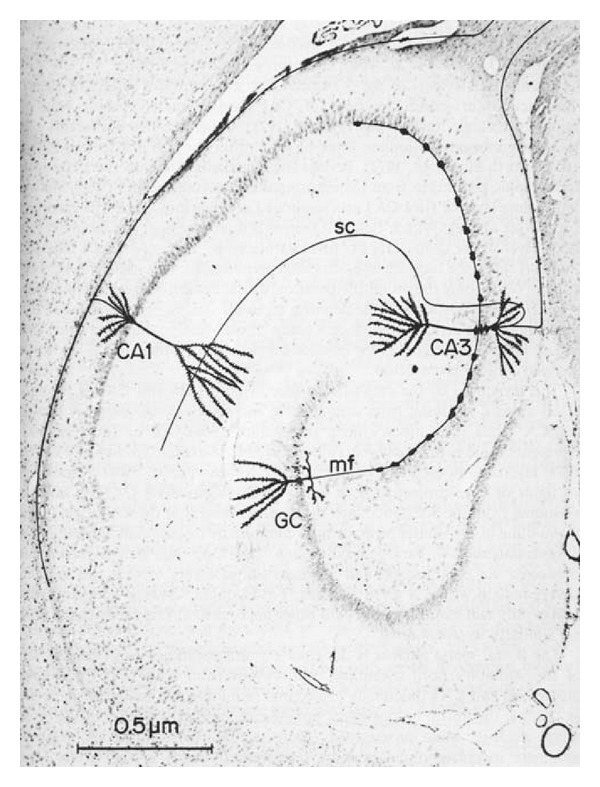
Hippocampal circuitry. Representative morphology of CA1 and CA3 pyramidal cells and a dentate granule cell (GC). The axon of the granule cell gives off collaterals in the hilus and a long branch (mf) extends into the CA3 field as a mossy fiber that contacts heavy thorns on the CA3 pyramidal cell. The axon of the CA3 pyramidal cell gives off a Shaffer collateral (SC) in the stratum oriens which ascends to the stratum lacunosum moleculare. The axon of the CA1 pyramidal cell bifurcates into long extensions within the white matter. Republished from [57] with permissions from Elsevier and the author.

Nearly all neocortical-hippocampal connections are indirect, being relayed via the perforant pathway of the entorhinal cortex. Stimulation of rat and human dentate gyrus granule cells by the perforant pathway activates a sequence of intrinsic connections within the hippocampal formation (Figure 8) that allows the hippocampus to remain informed of ongoing neocortical sensory activity [56, 57]. Stimulation of this circuitry culminates in hippocampal and subicular output back to the deeper part of the entorhinal cortex, reciprocating the perforant pathway [64]. Human hippocampal and subicular neurons project heavily back to layer IV pyramidal neurons and large multipolar neurons of the deep entorhinal cortex that in turn project widely to association cortices and limbic structures. Layers IV, V, and VI of the entorhinal cortex are present in rats but are less well-defined than in humans [58]. In general, major cytoarchitectonic features of the entorhinal cortex are well preserved in brains of aged nondemented human controls [65] and rat controls (Walton, personal observations), even in the oldest old.

The hippocampal formation of healthy humans is also larger and much more complex than in rats. CA1, CA2, and CA3 hippocampal fields of the normal rat appear in optical sections as a distinct, compact, and continuous band of cells, several layers in width, whereas in equivalent sections of the human hippocampus these cells are more widely spaced and their organization is less clear.

## 7. Pathological Changes in the Entorhinal Cortex and Perforant Pathway of Rats and Humans

 Brains of humans with AD show strikingly similar outcomes to brains of rats subjected to transection of their angular bundle or extensive transection of the perforant pathway [56, 63, 66–69]. In both species, terminals are drastically reduced in the outer two-thirds of the dentate molecular layer where most axons of the perforant pathway normally terminate. Interruption of the perforant pathway by bilateral transection also results in pronounced memory impairment in rats [11, 12, 67]. The consequences of perforant path transection in rats are also very similar to those that occur in humans who have had their entorhinal cortex and hippocampus bilaterally destroyed by surgery [70], and in victims of herpes simplex encephalopathy [71]. 

An invariant feature of AD is that the normal cytoarchitectonic pattern of the intermediate and lateral regions of the entorhinal cortex is markedly altered by NFTs, prominent in layers II and IV and to a lesser extent in superficial parts of layer III [56, 63]. By contrast, the smaller cells of layers V and VI are relatively spared. The entorhinal cortex is the most affected region of the AD brain. It has by far more NFTs than any other of Brodmann's areas. NFTs in this region of AD brains are usually extracellular or “ghost” NFTs that have outlived their host cells [56].

Intraneuronal Al is involved in the formation and growth of human NFTs [72]. Consequently, large NFTs can be regarded as markers of pyramidal cells with high-stage Al accumulation. High-stage nuclear Al accumulation occurs in the same cell types (stellate and pyramidal cells) in equivalent brain regions of the rat model as those where NFTs form in AD cases.

Previously, the selective vulnerability of entorhinal, hippocampal, and cortical cells to damage in AD was a mystery [56]. This animal model demonstrates that Al derived from dietary exposure at human-relevant levels preferentially accumulates in the cells of origin for the perforant pathway and other large AD-vulnerable pyramidal cells. Thus, the translational animal model helps to explain how the perforant path becomes interrupted in AD.

In AD, a conspicuous layer of neuritic plaques forms down the center of the molecular layer of the dentate gyrus [63], precisely in the termination zone of perforant pathways that have NFT-damaged cells of origin. Hyman et al. [63] observed that this finding is consistent with the hypothesis that neuritic plaques represent degenerating terminals [73].

Compensatory sprouting in response to this damage and reinnervation by less damaged entorhinal cortical cells is likely to continue for some time but this type of repair decreases with age [9] and eventually exhausts in AD [48, 65]. This may explain why cognitive deterioration became apparent in most of the susceptible rats to chronic Al neurotoxicity around 27-28 months of age. The perforant path aspect of AD provides a structural basis for early memory changes that occur in AD. Confusion and inability to recall new episodes occur relatively early in the course of AD and directly affect cognition [56]. Widespread changes occur in the brain as AD continues to progress. This is consistent with Al levels increasing over time to neurotoxic thresholds in other AD-vulnerable brain regions as a result of continuing human exposure to Al in foods, water, and other sources.

New imaging techniques are being applied to the brain. The deteriorating perforant pathway in Alzheimer brains can now be observed with diffusion tensor imaging [74]. *Ex vivo* imaging is currently being used to visualize the perforant pathway in unsectioned human brain. The goal is to develop an *in vivo* imaging technique for examining change in the perforant pathway of living humans for diagnostic purposes [75].

## 8. Pathological Changes in the Hippocampal Formation of Humans with AD and Rats with Chronic Aluminum Neurotoxicity

Destruction of the perforant pathway severely deafferents the hippocampal formation [76]. Specific pathological changes occur in the deafferented AD hippocampal formation. Pyramidal cells in the CA1/subicular zone are prone to NFT formation. Some severely affected AD cases show mostly ghost NFTs in the CA1 hippocampal field, indicating the CA1 is virtually destroyed. Pyramidal cells in other hippocampal fields are shrunken with stage V Al staining, appearing abnormal but viable. Most AD cases generally have less severe pathology. Damage to the subicular and CA1 neurons, that normally give rise to the major cortical and subcortical output of the hippocampal formation, compromises output from the hippocampal formation [76]. This results in disconnection of hippocampal efferents including the strong subicular projection to layer IV of the entorhinal cortex that normally has widespread neocortical connections.

Brains of all aged rats exhibited some cells with stage IV Al accumulation. Cognition remains intact as long as cells with stage IV Al accumulation are few and scattered. The entorhinal cortex of rats that developed cognitive deterioration contained a critical proportion of cells of origin for the perforant pathway damaged by stage IV Al accumulation and microtubule depletion, leaving insufficient numbers of healthy cells to effectively convey cortical information to the hippocampal formation. The second component of cognitive deterioration is the presence of at least one substantial lesion consisting of stage IV Al-rich microtubule-depleted pyramidal cells in the hippocampal CA1/subicular zone that fail to reciprocate output from the hippocampal formation back to deeper layers of the entorhinal cortex and from there to the neocortex. The CA1 lesion exemplified in Figure 4 extended throughout the entire rostrocaudal axis of the hippocampus. The rats that retained normal cognition were free of such lesions and their entorhinal cortex had smaller proportions of Al-affected cells. 

Subsequent examination of AD hippocampal sections also revealed discrete lesions in the hippocampal CA1 field [31]. In this case, the lesions consisted mainly of NFT-containing cells. Additional cells showed various stages of nuclear Al accumulation.

Other investigators have observed that NFT-containing cells in AD neocortical regions are also in the form of lesions [77–79]. At first these cells appear sporadically. As cells with NFTs continue to increase in number, they take the form of cell clusters. Eventually, as more cells between the clusters are recruited into the lesions, they appear in the form of cell bands [78]. We have also made observations of Al-rich lesions in neocortical regions that normally communicate with the entorhinal cortex in rats with cognitive deterioration [31]. 

Thus, cognitive deterioration in the Al-inducible rat model for chronic Al neurotoxicity/AD and AD in humans both involve Al-induced damage to the cells of origin for the perforant path projection to the hippocampal formation together with CA1 or subicular lesions in the hippocampal formation. These profound pathological changes effectively disconnect the hippocampal formation from limbic and association cortices [63]. Cortical regions depend upon the hippocampal formation for memory consolidation. The structural changes that occur in these AD brain regions preclude the normal acquisition of episodic or contextual knowledge [63], undoubtedly contributing to the cognitive deterioration in AD.

The translational animal model shows that stage IV Al accumulation in the entorhinal cortical cells of origin for the perforant pathway and in the lesions of CA1 or subicular cells, exhibiting microtubule depletion and dendritic dieback, is sufficient to result in observable cognitive deterioration.

Sources of neocortical and subcortical input to the entorhinal cortex are also targets for AD pathology, eliminating nearly all potential redundancy [56]. Neocortical layers III and V are particularly affected by NFT formation. These changes are uncharacteristic of other dementias such as those associated with Huntington's disease and multi-infarct dementia [63].

## 9. Physiological Similarities between Rats with Chronic Al Neurotoxicity and Humans with AD

Rats that consumed Al at the highest level had proportionately higher serum Al levels and more within-group variability than the other two groups. Specifically, those that consumed the highest Al dose level had a significantly higher mean serum Al level than the low Al dose group (*P* < 0.05). Also, the rats that developed cognitive deterioration in old age generally had higher serum Al levels than rats that remained cognitively intact (*P* < 0.01) [8].

This is also the case for humans that develop AD in old age. Six out of seven studies have shown that AD patients have higher serum or plasma Al levels than nondemented controls [80–85]. The seventh was a small study with low statistical power [86]. AD patients also absorbed 1.4 times more ^26^Al than age-matched controls from a standardized ^26^Al dose [87]. Similarly, patients with Down's syndrome (DS) absorbed ^26^Al six times more efficiently than age-matched controls after consuming a standardized ^26^Al dose at the dietary level and four times more efficiently from a pharmacological (antacid) Al level [88]. Brains of DS cases had Al levels comparable to those in AD brains but at earlier ages [51]. DS patients have been regarded by some as a human model for AD on the basis that they typically develop AD-type neuropathology by age 50 [89] and have a high risk for AD-type dementia [90]. Highly efficient Al absorption may account for the early appearance of AD characteristics.

## 10. Involvement of Aluminum with Other Dementias

Outcomes of Al exposure depend on the age and physical condition of the subject, the form or species of Al, the size of Al dose(s), and the frequency of exposure (whether chronic, acute or subacute). 

Al is a dementia-causing metal. Al causes dialysis encephalopathy [91], a dementia that is generally fatal unless the affected renal patients are treated and controlled with Al chelation [92]. Al has also been implicated in dementias associated with occupational Al exposure, including Balint's syndrome [93] and in the amyotrophic lateral sclerosis with parkinsonism dementia of Guam (ALS/PD) [94]. Al caused cognitive impairment in Canadian miners who inhaled Macintyre powder (pulverized Al and Al hydroxide) over an extended time to avoid lung silicosis [95]. ALS/PD and AD share common pathological characteristics, both showing hippocampal granulovacuolar degeneration (GVD) and Al-containing NFTs in the brains of affected patients [30, 96, 97]. Al has been regarded as a possible cause of AD since 1973 when it was first shown that specific AD brain regions contain higher Al levels than controls [98].

## 11. Involvement of Aluminum in Alzheimer Neuropathological Hallmarks

### 11.1. Neurofibrillary Tangles (NFTs)

Al induces hyperphosphorylation of tau and other neural proteins by inhibiting protein phosphatases [39, 99–101]. Protein phosphatase 2A (PP2A) is the main phosphatase that dephosphorylates tau in mammalian brain tissue [102]. PP2A activity is inhibited, and gives rise to hyperphosphorylated tau, in AD brain tissue and in the cortex and hippocampus of rats with Al-inducible cognitive deterioration [39, 103]. AD involves a massive accumulation of hyperphosphorylated tau and a severe loss of normal tau in cortical tissue [104]. The same unusual phenomenon occurs in brains of renal dialysis patients who have had high Al exposure [105].

Al colocalizes with hyperphosphorylated tau in pretangle neurons of AD brains, forming cytoplasmic pools of an Al/hyperphosphorylated tau complex [72]. Al/hyperphosphorylated tau molecules polymerize within the cytoplasmic pools, giving rise to the filaments that constitute NFTs. Al and hyperphosphorylated tau also coparticipate in NFT growth as Al uptake into human hippocampal and cortical pyramidal cells continues over time [72].

AD cells that contain NFTs exhibit less nuclear Al staining than cells that stain positively for Al without NFTs. These neuropathological data suggest that the NFTs that form initially protect cells by retaining Al in the cytoplasm and slowing Al entry into the nucleus [72]. However, large NFTs generally displace the nucleus to the cell periphery and, if sufficiently large, can eventually enucleate the cell [30]. Hence, NFT overgrowth is probably responsible for much of the cell death that occurs in the AD entorhinal cortex, CA1 field, subiculum, and other brain regions where NFTs form.

Histological and immunocytochemical antibody stains reveal Al in human NFTs [30, 31, 106, 107]. Various instrumental techniques have also been used to demonstrate Al in NFTs [98, 108, 109]. Such studies have shown that NFTs contain up to 300 *μ*g Al/g tissue [110].

NFT formation is species specific. NFTs develop without experimental intervention in the brains of aged cats [111]. Al injection into the lateral ventricles of cats rapidly induces the formation of NFTs accompanied by disturbance in brain electrical activity and alterations in acquisition and retention tasks. Cat pyramidal cells become microtubule depleted as they develop NFTs [112].

NFT formation without experimental intervention has yet to be reported in brains of aged rabbits. Al injection into the lateral ventricles of rabbit brain has been observed to induce NFTs in pyramidal cells of brain regions analogous to the same regions where NFTs form in AD-affected humans and this is often accompanied by encephalopathy [35]. In contrast, Al injection into the lateral ventricles of rat brains impairs performance on behavioral tasks in the absence of NFT formation [113]. These differences between cats, rabbits, and rats illustrate the species specificity of NFTs.

Rabbit NFTs appear similar to human NFTs when examined by optical microscopy but are biochemically and ultrastructurally different. This biochemical difference is likely to be a species effect but could also be a temporal effect since Al-induced NFTs are newly formed whereas human NFTs are generally long standing. The biochemical composition of rabbit NFTs changes within days of formation, acquiring tau and other proteins that increase their similarity to human NFTs [114, 115].

Much discussion has centered around Al-induced NFTs being straight filaments and AD NFTs consisting of paired helical filaments (PHFs). Actually, this appears to be a misnomer because the filaments that comprise human NFTs are single filaments with a twisted ribbon structure [116, 117]. Some are straight while others are partly twisted and partly straight. Hence, the structural differences of NFTs in different species may amount to sequence differences in the human, rabbit, and cat forms of tau.

### 11.2. Amyloid Plaques and Presenilins

Al is also involved in the formation of amyloid plaques. Nanomolar amounts of Al upregulate gene expression for amyloid precursor protein in human neural cells [118, 119]. APP mRNA and protein are also upregulated in brains of rats with cognitive deterioration that were chronically exposed to human-relevant Al levels [40] and APP-related pathology was evident in brains of rats given intracerebral Al injections [120]. Furthermore, Al produces effects that divert APP metabolism from its nonamyloidogenic pathway to its amyloidogenic pathway, resulting in the formation of *β*-amyloid oligomers, fibrils, and plaques [121–128].

Al also increases amyloidogenesis in transgenic mice that express a mutant human form of APP. APP-transgenic mice fed a diet supplemented with Al for one year exhibited oxidative damage accompanied by more numerous and larger amyloid plaques in their brains than a transgenic cohort without Al supplementation [129].

In addition, an aberrant variant of presenilin-2 (PS2V) increases production of intracellular *β*-amyloid_1-42_ and *β*-amyloid_1-40_ by impairing the signaling pathway for the unfolded protein response and interfering with APP maturation [130]. This variant occurs almost exclusively in brains of humans with sporadic AD. PS2V was identified in 10/10 brains with sporadic AD and only at a low level in 1/10 brains from controls [131]. PS2V is induced by hypoxia and oxidative stress, suggesting it may be inducible by one or more metal prooxidants. Al, which is a prooxidant in its own right as well as synergistically with iron, was found to be the only metal capable of inducing PS2V formation [132].

### 11.3. Granulovacuolar Degeneration (GVD)

GVD is the third most prominent AD hallmark in the hippocampus. Al is the only known toxic agent that can experimentally induce hippocampal GVD. Rats develop hippocampal GVD after receiving repeated intraperitoneal injections of Al [133, 134]. GVD also occurred in the hippocampus of the translational animal model with cognitive deterioration that chronically consumed Al at human-relevant dietary Al levels [39]. Thus, substantial evidence attests to amyloid plaques, NFTs, and GVD as all being by-products of Al activity in the brain.

### 11.4. Other AD Features

Al accumulation in pyramidal neurons disrupts their calcium metabolism in ways remarkably similar to those that occur with aging and AD (reviewed in [128]). Al accumulation in cells disrupts the calcium phosphoinositide signaling pathway and the process of calcium signaling, interferes with calcium participation in long-term potentiation, and inhibits calcium-regulatory enzymes in a dose-dependent manner. 

Al also disrupts iron metabolism in ways similar to those that occur in AD. In AD, iron levels are elevated in pyramidal cells of AD-vulnerable brains regions, particularly in the hippocampus, amygdala, and inferior parietal cortex [135]. Iron regulatory protein-2 (IRP-2) is stabilized in AD so cells behave as though they are permanently iron deficient [136]. This signals the cell to continue synthesizing transferrin receptors, leading to abnormally high levels of free iron in the cells and increasing oxidative damage. 

The Al^3+^ ion is almost the same size as the Fe^3+^ ion [137] and occupies iron sites in transferrin (the plasma iron transport protein) as Al circulates through the body [138, 139]. This facilitates Al uptake into endothelial cells [140] and from there into brain cells [141] via transferrin receptors. Al also enters cells by a transferrin-independent uptake mechanism [142]. Intraneuronal Al stabilizes the expression of IRP-2 in pyramidal cells by preventing its breakdown [143]. Thus, the IRP-2 continues to promote the synthesis of transferrin receptors and iron uptake into the cells as in AD. 

Al thereby increases iron levels in cultured neural cells [144] as well as in the hippocampus, frontal cortex, and temporal cortex of Al-loaded rats [145]. Experimental animal studies have shown that Al accumulation in cholinergic neurons, or in neurons of the locus coeruleus and dorsal raphe nucleus, is accompanied by decreased concentrations of their respective neurotransmitters; namely, acetylcholine, norepinephrine (noradrenaline) and serotonin [146, 147].

Thus, chronic Al neurotoxicity influences all major neuropathological features of AD. Ganrot expressed essentially the same conclusion a quarter of a century ago [148]. Importantly, Al preferentially accumulates in the same regions of human and animal brains as those that are affected in AD. This distribution also occurs in brains of rabbits with acute Al neurotoxicity following stereotaxic Al injection into brain ventricles [35], in brains of chronic dialysis patients with subacute Al neurotoxicity, [149, 150] and in brains of rats with chronic Al neurotoxicity from ingesting Al at human-relevant levels [8].

## 12. Background Reasons Why Rats with Chronic Al Neurotoxicity Are a Useful Translational Model for AD

An epidemiological study to assess the risk of AD from total dietary Al exposure from food, drinking water, and Al additives has yet to be carried out. Studies that only consider a single source of Al exposure are susceptible to significant confounding. It would be impractical and probably unethical to attempt to perform a longitudinal randomized controlled trial that intentionally assigns groups of humans to high levels, as well as lower levels, of a neurotoxicant such as Al, over a long period of time, to learn where the neurotoxin deposits in the brain as the subjects age and to examine the long-term consequences of such treatment.

Instead, a surrogate is clearly needed. Al intake was carefully controlled and monitored in this translational animal model for human total dietary Al exposure. It is also more convenient to carry out such longitudinal studies in animals that age more rapidly and have a naturally short life span. The chronic Al neurotoxicity model permits interventional studies to slow, arrest, and possibly reverse AD and to determine the stages when the relevant action may be best implemented.

## 13. Conclusions and Recommendations

This translational model demonstrates that chronic ingestion of Al, in amounts ingested by Americans from their food, water, and Al additives, is sufficient to induce AD-type cognitive deterioration in animals by old age. The time required for Al to gradually accumulate in neurons to a neurotoxic threshold can account for the long prodromal phase in this animal model and possibly in AD as well. The slow accumulation of Al in brain cells may also explain why AD generally affects older individuals.

It is apparent from Al levels in blood samples that some rats and humans absorb Al more efficiently than others. The model indicates (1) that a minority of individuals (approximately 20% of rats under the present circumstances) are susceptible to cognitive deterioration after ingesting a relatively low concentration of Al that the others can tolerate without obvious adverse effects and (2) that other individuals have high serum Al levels because they consume too many foods with Al additives. The model indicates that establishment of a standardized diagnostic test for Al absorption would be useful to determine individual susceptibility to chronic Al neurotoxicity so they could exercise prudcnt avoidance of Al exposure.

The neuropathology of this translational animal model provides parallels with AD features. In particular, Al accumulation in the entorhinal cortical cells of origin for the perforant pathway, and damage to these cells and the cells of the hippocampal CA1 field and subiculum, results in isolation of the hippocampal formation from the neocortex. 

The fundamental similarities between clinical and neuropathological evidence from aged humans with AD and this inducible animal model that develops cognitive deterioration in old age lead to the conclusion that AD is a form of chronic Al neurotoxicity that becomes evident as Al accumulation in the entorhinal cortical cells of origin for the perforant pathway and the hippocampal CA1 field surpasses a neurotoxic threshold, causing communication failure between these brain regions that are pivotal to memory and learning.

This study has given rise to the following recommendations to assist with the prevention, diagnosis, and treatment of AD:

(I) The translational model of itself points to an array of experimental uses which will assist in a more rapid understanding of AD diagnosis, pathology, treatment, and prevention.

- Determination of parameters for serum/plasma tests for Al at different ages: the translational animal model could be used to probe why some individuals routinely absorb Al more efficiently than others.
- Development of methods using instrumentation such as the diffusion tension imaging system that can visualize deteriorative change in the perforant pathway: the animal model could serve as an intermediate subject for an imaging method lying between the present histopathology of AD autopsy specimens and detailed viewing of the relevant brain areas of living patients, with a view to diagnosing AD well before the occurrence of ventricular enlargement and widespread cortical atrophy.
- Variation of the Al dose level could be made in future experiments by increasing the total Al level up to 10 mg/kg bw/day, or by starting treatment several months earlier, with a view to producing cognitive damage in all animals rather than in 70% as reported in our larger study [8]. With this foundation, interventional studies could be developed for the prevention and delay of otherwise certain cognitive deterioration, and for remission and recovery at early stages of damage.
- By way of example, desferrioxamine and/or newer generation Al chelating agents could be tested to determine how far cognitive deterioration can progress and still be treatable by Al chelation and removal from the brain.

(II) As to immediate action, at the human level, the following can be considered and implemented appropriately.

- Provision to consumers of more complete information as to the presence, type, and amounts of Al contained in commercially prepared foods and drinking water to enable consumers to make informed choices.
- Establishment of a routine standardized blood test designed to determine risk for AD/chronic Al neurotoxicity based on the individual's efficacy for Al absorption. The testing protocol would need to consider many factors and, if feasible, include a challenge method, given that an individual's response to Al generally peaks within 30–60 minutes depending on the form of Al ingested. The test history could form a basis for clinical advice to those at particular risk and need the benefit of an informed choice as to ingestion of foods and water containing Al additives, vaccinations containing Al adjuvants, and other miscellaneous sources of exposure to Al.

## References

[1] Price M, Jackson J http://www.alz.co.uk/research/files/World%20Alzheimer%20Report.pdf.

[2] Morris JC (1994). Differential diagnosis of Alzheimer’s disease. *Clinics in Geriatric Medicine*.

[3] Humphry GM (1889). *Old Age: The Results of Information Received Respecting Nearly
Nine Hundred Persons Who Had Attained the Age of Eighty Years, Including Seventy-Four
Centenarians*.

[4] Henderson AS (1986). The epidemiology of Alzheimer’s disease. *British Medical Bulletin*.

[5] Blansjaar BA, Thomassen R, Van Schaick HW (2000). Prevalence of dementia in centenarians. *International Journal of Geriatric Psychiatry*.

[6] Alzheimer A (1907). Ueber eine eigenartige Erkrankung der Hirnrinde. *Zentralblatt fur Nevenheilkunde Psychiatrie*.

[7] Walton JR (2007). A longitudinal study of rats chronically exposed to aluminum at human dietary levels. *Neuroscience Letters*.

[8] Walton JR (2009). Functional impairment in aged rats chronically exposed to human range dietary aluminum equivalents. *NeuroToxicology*.

[9] Hinds JW, McNelly NA (1977). Aging of the rat olfactory bulb: growth and atrophy of constituent layers and changes in size and number of mitral cells. *Journal of Comparative Neurology*.

[10] Walton JR (1982). The role of limited cell replicative capacity in pathological age change. A review. *Mechanisms of Ageing and Development*.

[11] Loesche O, Steward J (1977). Behavioral correlates of denervation and
reinnervation of the hippocampal formation of the rat: recovery of alternation
performance following unilateral entorhinal cortical lesions. *Brain Research Bulletin*.

[12] Steward O, Loesche J, Horton WC (1977). Behavioral correlates of denervation and reinnervation of the hippocampal formation of the rat: open field activity and cue utilization following bilateral entorhinal cortex lesions. *Brain Research Bulletin*.

[13] Greger JL (1993). Aluminum metabolism. *Annual Review of Nutrition*.

[14] Everitt AV (1991). Ageing rat colonies at the University of Sydney. *Proceedings of the Australian Association for Gerontology*.

[15] McLachlan DR (1995). Aluminium and the risk for Alzheimer’s disease. *Environmetrics*.

[16] Markesbery WR, Ehmann WD, Hossain TIM (1981). Instrumental neutron activation analysis of brain aluminum in Alzheimer disease and aging. *Annals of Neurology*.

[17] McDermott JR, Smith AI, Iqbal K, Wisniewski HM (1979). Brain aluminium in aging and Alzheimer disease. *Neurology*.

[18] Shimizu H, Mori T, Koama M, Sekiya M, Ooami H (1994). A correlative study of the
aluminum content and aging changes of the brain in non-demented elderly subjects. *Nippon Ronen Igakkai Zasshi*.

[19] Edwardson JA, Moore PB, Ferrier IN (1993). Effect of silicon on gastrointestinal absorption of aluminium. *The Lancet*.

[20] World Health Organization http://www.inchem.org/documents/jecfa/jecmono/v024je07.htm.

[21] Walton JR (2012). Evidence that total dietary aluminum ingestion is a major risk
factor for Alzheimer’s disease. *Current Inorganic Chemistry*.

[22] Walton J, Tuniz C, Fink D, Jacobsen G, Wilcox D (1995). Uptake of trace amounts of aluminum into the brain from drinking water. *NeuroToxicology*.

[23] Drüeke TB, Jouhanneau P, Banide H, Lacour B, Yiou F, Raisbeck G (1997). Effects of silicon, citrate and the fasting state on the intestinal absorption of aluminium in rats. *Clinical Science*.

[24] Zafar TA, Weaver CM, Martin BR, Flarend R, Elmore D (1997). Aluminum (26Al) Metabolism in Rats. *Experimental Biology and Medicine*.

[25] World Health Organization (2007). Evaluation of certain food additives and
contaminants in Sixty-seventh report of the joint FAO/WHO Expert Committee on
Food Additives. *WHO Technical Report Series*.

[26] Tomljenovic L, Shaw CA (2011). Aluminum vaccine adjuvants: are they safe?. *Current Medicinal Chemistry*.

[27] Redhead K, Quinlan GJ, Das RG, Gutteridge JMC (1992). Aluminium-adjuvanted vaccines transiently increase aluminium levels in murine brain tissue. *Pharmacology and Toxicology*.

[28] Anane R, Bonini M, Grafeille JM, Creppy EE (1995). Bioaccumulation of water soluble aluminium chloride in the hippocampus after transdermal uptake in mice. *Archives of Toxicology*.

[29] Lione A (1985). Aluminum toxicology and the aluminum-containing medications. *Pharmacology and Therapeutics*.

[30] Walton JR (2006). Aluminum in hippocampal neurons from humans with Alzheimer’s disease. *NeuroToxicology*.

[31] Walton JR (2009). Brain lesions comprised of aluminum-rich cells that lack microtubules may be associated with the cognitive deficit of Alzheimer’s disease. *NeuroToxicology*.

[32] McLachlan DRC, Dalton AJ, Kruck TPA (1991). Intramuscular desferrioxamine in patients with Alzheimer’s disease. *The Lancet*.

[33] McLachlan DRC, Smith WL, Kruck TP (1993). Desferrioxamine and Alzheimer’s disease: video home behavior assessment of clinical course and measures of brain aluminum. *Therapeutic Drug Monitoring*.

[34] Walton JR (2004). A bright field/fluorescent stain for aluminum: its specificity, validation, and staining characteristics. *Biotechnic and Histochemistry*.

[35] Kowall NW, Pendlebury WW, Kessler JB, Perl DP, Beal MF (1989). Aluminum-induced neurofibrillary degeneration affects a subset of neurons in rabbit cerebral cortex, basal forebrain and upper brainstem. *Neuroscience*.

[36] Braak H, Braak E (1991). Neuropathological stageing of Alzheimer-related changes. *Acta Neuropathologica*.

[37] Kovács T, Cairns NJ, Lantos PL (2001). Olfactory centres in Alzheimer's disease: olfactory bulb is involved in early Braak's stages. *NeuroReport*.

[38] Arnold SE, Hyman BT, Flory J, Damasio AR, Van Hoesen GW (1991). The topographical and neuroanatomical distribution of neurofibrillary tangles and neuritic plaques in the cerebral cortex of patients with Alzheimer’s disease. *Cerebral Cortex*.

[39] Walton JR (2007). An aluminum-based rat model for Alzheimer’s disease exhibits oxidative damage, inhibition of PP2A activity, hyperphosphorylated tau, and granulovacuolar degeneration. *Journal of Inorganic Biochemistry*.

[40] Walton JR, Wang MX (2009). APP expression, distribution and accumulation are altered by aluminum in a rodent model for Alzheimer’s disease. *Journal of Inorganic Biochemistry*.

[41] Baas PW (2002). Microtubule transport in the axon. *International Review of Cytology*.

[42] Gray EG, Paula-Barbosa M, Roher A (1987). Alzheimer’s disease: paired helical filaments and cytomembranes. *Neuropathology and Applied Neurobiology*.

[43] Hempen B, Brion JP (1996). Reduction of acetylated *α*-tubulin immunoreactivity in neurofibrillary tangle-bearing neurons in Alzheimer’s disease. *Journal of Neuropathology and Experimental Neurology*.

[44] Hatanpää K, Brady DR, Stoll J, Rapoport SI, Chandrasekaran K (1996). Neuronal activity and early neurofibrillary tangles in Alzheimer’s disease. *Annals of Neurology*.

[45] Scheibel ME, Lindsay RD, Tomiyasu U, Scheibel AB (1976). Progressive dendritic changes in the aging human limbic system. *Experimental Neurology*.

[46] Petit TL, Biederman GB, McCullen PA (1980). Neurofibrillary degeneration, dendritic dying back, and learning-memory deficits after aluminum administration: implications for brain aging. *Experimental Neurology*.

[47] Uemura E, Ireland WP (1985). Dendritic alterations in chronic animals with experimental neurofibrillary changes. *Experimental Neurology*.

[48] Flood DG, Coleman PD (1986). Failed compensatory dendritic growth as a pathophysiological process in Alzheimer’s disease. *Canadian Journal of Neurological Sciences*.

[49] Terry RD, Masliah E, Salmon DP (1991). Physical basis of cognitive alterations in Alzheimer’s disease: synapse loss is the major correlate of cognitive impairment. *Annals of Neurology*.

[50] Andrasi E, Pali N, Molnar Z, Kosel S (2005). Brain aluminum, magnesium and
phosphorus contents of control and Alzheimer-diseased patients. *Journal of Alzheimer’s Disease*.

[51] Crapper DR, Krishnan SS, Quittkat S (1976). Aluminium, neurofibrillary degeneration and Alzheimer’s disease. *Brain*.

[52] Krishnan SS, McLachlan DR, Krishnan B, Fenton SSA, Harrison JE (1988). Aluminum toxicity to the brain. *Science of the Total Environment*.

[53] Trapp GA, Miner GD, Zimmerman RL (1978). Aluminum levels in brain in Alzheimer’s disease. *Biological Psychiatry*.

[54] Xu N, Majidi V, Markesbery WR, Ehmann WD (1992). Brain aluminum in Alzheimer’s disease using an improved GFAAS method. *NeuroToxicology*.

[55] Van Hoesen GW, Pandya DN (1975). Some connections of the entorhinal (area 28) and perirhinal (area 35) cortices of the rhesus monkey. III. Efferent connections. *Brain Research*.

[56] Van Hoesen GW, Hyman BT, Damasio AR (1991). Entorhinal cortex pathology in Alzheimer’s disease. *Hippocampus*.

[57] Bayer SA, Paxinos G (1985). Hippocampal region. *The Rat Central Nervous System*.

[58] Insausti R (1993). Comparative anatomy of the entorhinal cortex and hippocampus in mammals. *Hippocampus*.

[59] Germroth P, Schwerdtfeger WK, Buhl EH (1991). Ultrastructure and aspects of functional organization of pyramidal and nonpyramidal entorhinal projection neurons contributing to the perforant path. *Journal of Comparative Neurology*.

[60] Beall MJ, Lewis DA (1992). Heterogeneity of layer II neurons in human entorhinal cortex. *Journal of Comparative Neurology*.

[61] Mikkonen M, Pitkänen A, Soininen H, Alafuzoff I, Miettinen R (2000). Morphology of spiny neurons in the human entorhinal cortex: intracellular filling with Lucifer Yellow. *Neuroscience*.

[62] Klink A, Alonso R (1997). Morphological characteristics of layer II projection
neurons in the rat medial entorhinal cortex. *Hippocampus*.

[63] Hyman BT, Van Hoesen GW, Kromer LJ, Damasio AR (1986). Perforant pathway changes and the memory impairment of Alzheimer’s disease. *Annals of Neurology*.

[64] Beckstead RM (1978). Afferent connections of the entorhinal area in the rat as demonstrated by retrograde cell-labeling with horseradish peroxidase. *Brain Research*.

[65] Flood DG, Buell SJ, Defiore CH, Horwitz GJ, Coleman PD (1985). Agerelated
dendritic growth in dentate gyrus of human brain is followed by regression in
the ‘oldest old’. *Brain Research*.

[66] Grandes P, Streit P (1991). Effect of perforant path lesion on pattern of glutamate-like immunoreactivity in rat dentate gyrus. *Neuroscience*.

[67] Skelton RW (1998). Modelling recovery of cognitive function after traumatic brain injury: spatial navigation in the Morris water maze after complete or partial transections of the perforant path in rats. *Behavioural Brain Research*.

[68] Geddes JW, Monaghan DT, Cotman CW (1985). Plasticity of hippocampal circuitry in Alzheimer’s disease. *Science*.

[69] Van Hoesen GW (1982). The primate parahippocampal gyrus: new insights regarding
its cortical connections. *Trends in Neurosciences*.

[70] Scoville WB, Milner B (1957). Loss of recent memory after bilateral hippocampal lesions. *Journal of Neurology, Neurosurgery, and Psychiatry*.

[71] Damasio AR, Eslinger PJ, Damasio H, Van Hoesen GW, Cornell S (1985). Multi-modal amnesic syndrome following bilateral temporal and basal forebrain
damage: the case of patient DRB. *Archives of Neurology*.

[72] Walton JR (2010). Evidence for participation of aluminum in neurofibrillary tangle formation and growth in Alzheimer’s disease. *Journal of Alzheimer’s Disease*.

[73] Wisniewski HM, Merz GS, Katzman R (1983). Neuritic and amyloid plaques in senile
dementia of the Alzheimer type. *Banbury Report 15: Biological
Aspects of Alzheimer’s Disease*.

[74] Salat DH, Tuch DS, van der Kouwe AJW (2010). White matter pathology isolates the hippocampal formation in Alzheimer’s disease. *Neurobiology of Aging*.

[75] Augustinack JC, Helmer K, Huber KE, Kakunoori S, Zöllei L, Fischl B (2010). Direct visualization of the perforant pathway in the human brain with ex vivo diffusion tensor imaging. *Frontiers in Human Neuroscience*.

[76] Hyman BT, Van Hoesen GW, Damasio AR, Barnes CL (1984). Alzheimer’s disease: cell-specific pathology isolates the hippocampal formation. *Science*.

[77] Pearson RCA, Esiri MM, Hiorns RW (1985). Anatomical correlates of the distribution of the pathological changes in the neocortex in Alzheimer disease. *Proceedings of the National Academy of Sciences of the United States of America*.

[78] Armstrong RA (1993). Is the clustering of neurofibrillary tangles in Alzheimer’s patients related to the cells of origin of specific cortico-cortical projections?. *Neuroscience Letters*.

[79] Esiri MM, Chance SA (2006). Vulnerability to Alzheimer’s pathology in neocortex: the roles of plasticity and columnar organization. *Journal of Alzheimer’s Disease*.

[80] Yates PO, Mann DMA, Kellett JM (1986). Aluminosilicates and Alzheimer’s disease. *The Lancet*.

[81] Van Rhijn A, Corrigan FM, Ward NI (1989). Serum aluminum in senile dementia of Alzheimer’s type and in multi-infarct dementia. *Trace Elements in Medicine*.

[82] Corrigan FM, Crichton JS, Van Rhijn AG, Skinner ER, Ward NI (1992). Transferrin, cholesterol and aluminium in Alzheimer’s disease. *Clinica Chimica Acta*.

[83] Naylor GJ, Smith AHW, McHarg A (1989). Raised serum aluminum concentration in Alzheimer’s disease. *Trace Elements in Medicine*.

[84] Zapatero MD, De Jalon AG, Pascual F, Calvo ML, Escanero J, Marro A (1995). Serum aluminum levels in Alzheimer’s disease and other senile dementias. *Biological Trace Element Research*.

[85] Roberts NB, Clough A, Bellia JP, Kim JY (1998). Increased absorption of aluminium from a normal dietary intake in dementia. *Journal of Inorganic Biochemistry*.

[86] Shore D, Millson M, Holtz JL, King SW, Bridge TP, Wyatt RJ (1980). Serum
aluminum in primary degenerative dementia. *Biological Psychiatry*.

[87] Moore PB, Day JP, Taylor GA, Ferrier IN, Fifield LK, Edwardson JA (2000). Absorption of aluminium-26 in Alzheimer’s disease, measured using accelerator mass spectrometry. *Dementia and Geriatric Cognitive Disorders*.

[88] Moore PB, Edwardson JA, Ferrier IN (1997). Gastrointestinal absorption of aluminum is increased in Down’s syndrome. *Biological Psychiatry*.

[89] Mann DMA, Prasher VP (2006). Neuropathology of Alzheimer’s disease in Down syndrome. *Down Syndrome and Alzheimer’s Disease*.

[90] Ropper AH, Williams RS (1980). Relationship between plaques, tangles, and dementia in Down syndrome. *Neurology*.

[91] Alfrey AC, LeGendre GR, Kaehny D (1976). The dialysis encephalopathy syndrome. Possible aluminium intoxication. *New England Journal of Medicine*.

[92] Ackrill P, Day JP (1993). The use of desferrioximine in dialysis-associated aluminium disease. *Contributions to Nephrology*.

[93] Kobayashi S, Hirota N, Saito K, Utsuyama M (1987). Aluminum accumulation in tangle-bearing neurons of Alzheimer’s disease with Balint’s syndrome in a long-term aluminum refiner. *Acta Neuropathologica*.

[94] Yasui M, Yase Y, Ota K, Garruto RM (1991). Aluminum deposition in the central nervous system of patients with amyotrophic lateral sclerosis from the Kii Peninsula of Japan. *NeuroToxicology*.

[95] Rifat SL, Eastwood MR, Mclachlan DRC, Corey PN (1990). Effect of exposure of miners to aluminium powder. *The Lancet*.

[96] Simchowicz T (1914). La maladie d’Alzheimer et son rapport avec la démence sénile. *L’Encéphale*.

[97] Hirano A, Kurland LT, Krooth RS, Lessell S (1961). Parkinsonism-dementia complex, an endemic disease on the island of guam: I. Clinical features. *Brain*.

[98] Crapper DR, Krishnan SS, Dalton AJ (1973). Brain aluminum distribution in Alzheimer’s disease and experimental neurofibrillary degeneration. *Science*.

[99] Yamamoto H, Saitoh Y, Yasugawa S, Miyamoto E (1990). Dephosphorylation of tau
factor by protein phosphatase 2A in synaptosomal cytosol fractions, and inhibition by
aluminum. *Journal of Neurochemistry*.

[100] Amador FC, Henriques AG, Da Cruz E Silva OAB, Da Cruz E Silva EF (2004). Monitoring protein phosphatase 1 isoform levels as a marker for cellular stress. *Neurotoxicology and Teratology*.

[101] Cordeiro JM, Silva VS, Oliveira CR, Gonçalves PP (2003). Aluminium-induced impairment of Ca^2+^ modulatory action on GABA transport in brain cortex nerve terminals. *Journal of Inorganic Biochemistry*.

[102] Goedert M, Jakes R, Qi Z, Wang JH, Cohen P (1995). Protein phosphatase 2A is
the major enzyme in brain that dephosphorylates *τ* protein phosphorylated by prolinedirected
protein kinases or cyclic AMP-dependent protein kinase. *Journal of Neurochemistry*.

[103] Gong CX, Shaikh S, Wang JZ, Zaidi T, Grundke-Iqbal I, Iqbal K (1995). Phosphatase activity toward abnormally phosphorylated *τ*: decrease in Alzheimer disease brain. *Journal of Neurochemistry*.

[104] Shin R-W, Iwaki T, Kitamoto T, Sato Y, Tateishi J (1992). Massive
accumulation of modified tau and severe depletion of normal tau characterize the
cerebral cortex and white matter of Alzheimer’s disease. *American Journal of
Pathology*.

[105] Harrington CR, Wischik CM, McArthur FK, Taylor GA, Edwardson JM, Candy JA (1994). Alzheimer’s disease-like changes in tau protein processing:
association with aluminium accumulation in brains of renal diaysis patients. *The Lancet*.

[106] Levy R, Shohat L, Solomon B (1998). Specificity of an anti-aluminium monoclonal antibody toward free and protein-bound aluminium. *Journal of Inorganic Biochemistry*.

[107] Solomon B, Koppel R, Jossiphov J (2001). Immunostaining of calmodulin and aluminium in Alzheimer’s disease-affected brains. *Brain Research Bulletin*.

[108] Perl DP, Brody AR (1980). Alzheimer’s disease: X-ray spectrometric evidence of aluminum accumulation in neurofibrillary tangle-bearing neurons. *Science*.

[109] Good PF, Perl DP, Bierer LM, Schmeidler J (1992). Selective accumulation
of aluminum and iron in the neurofibrillary tangles of Alzheimer’s disease: a laser
microprobe [LAMMA] study. *Annals of Neurology*.

[110] Perl PF, Good DP (1987). The association of aluminium, Alzheimer’s disease
and neurofibrillary tangles. *Journal of Neural Transmission*.

[111] Head E, Moffat K, Das P (2005). *β*-Amyloid deposition and tau phosphorylation in clinically characterized aged cats. *Neurobiology of Aging*.

[112] Crapper DR, Dalton AJ (1973). Aluminum induced neurofibrillary degeneration, brain electrical activity and alterations in acquisition and retention. *Physiology and Behavior*.

[113] King GA, DeBoni U, Crapper DR (1975). Effect of aluminum upon conditioned
avoidance response acquisition in the absence of neurofibrillary degeneration. *Pharmacology, Biochemistry and Behavior*.

[114] Savory J, Huang Y, Herman MM, Reyes MR, Wills MR (1995). Tau
immunoreactivity associated with aluminum maltolate-induced neurofibrillary
degeneration in rabbits. *Brain Research*.

[115] Huang Y, Herman MM, Liu J, Katsetos CD, Wills MR, Savory J (1997). Neurofibrillary lesions in experimental aluminum-induced encephalopathy and Alzheimer’s disease share immunoreactivity for amyloid precursor protein, A*β*, *α*1-antichymotrypsin and ubiquitin-protein conjugates. *Brain Research*.

[116] Ruben GC, Wang JZ, Iqbal K, Grundke-Iqbal I (2005). Paired helical filaments (PHFs) are a family of single filament structures with a common helical turn period: negatively stained PHF imaged by TEM and measured before and after sonication, deglycosylation, and dephosphorylation. *Microscopy Research and Technique*.

[117] Wischik CM, Crowther RA, Stewart M, Roth M (1985). Subunit structure of paired helical filaments in Alzheimer’s disease. *Journal of Cell Biology*.

[118] Lukiw WJ (2004). Gene expression profiling in fetal, aged, and Alzheimer hippocampus: a continuum of stress-related signaling. *Neurochemical Research*.

[119] Alexandrov PN, Zhao Y, Pogue AI (2005). Synergistic effects of iron and aluminum on stress-related gene expression in primary human neural cells. *Journal of Alzheimer’s Disease*.

[120] Shigematsu PL, McGeer K (1992). Accumulation of amyloid precursor protein
in damaged neuronal processes and microglia following intracerebral administration
of aluminium salts. *Brain Research*.

[121] Exley C, Price NC, Kelly SM, Birchall JD (1993). An interaction of *β*-amyloid with aluminium in vitro. *FEBS Letters*.

[122] Kawahara M, Muramoto K, Kobayashi K, Mori H, Kuroda Y (1994). Aluminum
promotes the aggregation of Alzheimer’s amyloid beta-protein in vitro. *Biochemical
and Biophysical Research Communications*.

[123] Kawahara M, Kato M, Kuroda Y Y (2001). Effects of aluminum on the
neurotoxicity of primary cultured neurons and on the aggregation of beta-amyloid
protein. *Brain Research Bulletin*.

[124] House E, Collingwood J, Khan A, Korchazkina O, Berthon G, Exley C (2004). Aluminium, iron, zinc and copper influence the in vitro formation of amyloid fibrils
of Abeta_42_ in a manner which may have consequences for metal chelation therapy in
Alzheimer’s disease. *Journal of Alzheimer’s Disease*.

[125] Banks WA, Niehoff ML, Drago D, Zatta P (2006). Aluminum complexing enhances amyloid *β* protein penetration of blood-brain barrier. *Brain Research*.

[126] Rodella LF, Ricci F, Borsani E (2008). Aluminium exposure induces Alzheimer’s disease-like histopathological alterations in mouse brain. *Histology and Histopathology*.

[127] Drago D, Bolognin S, Zatta P (2008). Role of metal ions in the abeta
oligomerization in Alzheimer’s disease and in other neurological disorders. *Current
Alzheimer Research*.

[128] Walton JR (2012). Aluminum disruption of calcium homeostasis and signal
transduction resembles change that occurs in aging and Alzheimer’s disease. *Journal
of Alzheimer’s Disease*.

[129] Pratico D, Uryu K, Sung S, Tang S, Trojanowski JQ, -Y Lee VM (2002). Aluminum modulates brain amyloidosis through oxidative stress in APP transgenic
mice. *FASEB Journal*.

[130] Sato N, Hori O, Yamaguchi A (1999). A novel presenilin-2 splice variant in human Alzheimer’s disease
brain tissue. *Journal of Neurochemistry*.

[131] Sato N, Imaizumi K, Manabe T (2001). Increased production
of *β*-amyloid and vulnerability to endoplasmic reticulum stress by an aberrant spliced
form of presenilin 2. *Journal of Biological Chemistry*.

[132] Matsuzaki S, Manabe T, Katayama T (2004). Metals accelerate production of the aberrant splicing isoform of the presenilin-2. *Journal of Neurochemistry*.

[133] Sun X, Liu Z, Zhang X, Zhang Z (1999). Effects of aluminum on the number of
neurons [sic] granulovacuolar degeneration in rats. *Journal of Hygiene Research*.

[134] Miu AC, Andreescu CE, Vasiu R, Olteanu AI (2003). A behavioral and histological study of the effects of long-term exposure of adult rats to aluminum. *International Journal of Neuroscience*.

[135] Deibel MA, Ehmann WD, Markesbery WR (1996). Copper, iron and zinc
imbalances in severely degenerated brain regions in Alzheimer’s disease: possible relation
to oxidative stress. *Journal of Neurological Sciences*.

[136] Smith MA, Wehr K, Harris PLR, Siedlak SL, Connor JR, Perry G (1998). Abnormal localization of iron regulatory protein in Alzheimer’s disease. *Brain Research*.

[137] Martin RB (1986). The chemistry of aluminum as related to biology and medicine. *Clinical Chemistry*.

[138] Day JP, Barker J, Evans LJA (1991). Aluminium absorption studied by 26Al tracer. *The Lancet*.

[139] Trapp GA (1983). Plasma aluminum is bound to transferrin. *Life Sciences*.

[140] Jeffries WA, Brandon MR, Hunt SV, Williams AF, Gatter KC, Mason DY (1984). Transferrin receptors on endothelium of brain capillaries. *Nature*.

[141] Roskams AJ, Connor JR (1990). Aluminum access to the brain: a role for transferrin and its receptor. *Proceedings of the National Academy of Sciences of the United States of America*.

[142] Oshiro S, Kawahara M, Mika S (1998). Aluminum taken up by transferrin-independent
iron uptake affects the iron metabolism in rat cortical cells. *Journal of
Biochemistry*.

[143] Yamanaka K, Minato N, Iwai K (1999). Stabilization of iron regulatory protein 2, IRP2, by aluminum. *FEBS Letters*.

[144] Abreo K, Abreo F, Sella ML, Jain S (1999). Aluminum enhances iron uptake and
expression of neurofibrillary tangle protein in neuroblastoma cells. *Journal of
Neurochemistry*.

[145] Ward RM, Zhang Y, Crichton RR (2001). Aluminum toxicity and iron
homeostasis. *Journal of Inorganic Biochemistry*.

[146] Beal MF, Mazurek MF, Ellison DW, Kowall NW, Solomon PR, Pendlebury WW (1989). Neurochemical characteristics of aluminum-induced neurofibrillary
degeneration in rabbits. *Neuroscience*.

[147] Julka D, Sandhir R, Gill KD (1995). Altered cholinergic metabolism in rat CHS
following aluminum exposure: implications on learning performance. *Journal of
Neurochemistry*.

[148] Ganrot PO (1986). Metabolism and possible health effects of aluminium. *Environmental
Health Perspectives*.

[149] Morris CM, Candy JM, Oakley AE (1989). Comparison of the regional distribution of transferrin receptors and aluminium in the forebrain of chronic renal dialysis patients. *Journal of the Neurological Sciences*.

[150] Edwardson JA, Ferrier I, McArthur FK, Nicolini M, Zatta PF, Corain B (1991). Alzheimer’s disease and the aluminium hypothesis. *Aluminum
in Chemistry Biology and Medicine. A Series of Advances*.

